# Reactive Oxygen Species-Based Nanomaterials for Cancer Therapy

**DOI:** 10.3389/fchem.2021.650587

**Published:** 2021-04-22

**Authors:** Yingbo Li, Jie Yang, Xilin Sun

**Affiliations:** ^1^National Health Commission and Chinese Academy of Medical Sciences Key Laboratory of Molecular Probe and Targeted Theranostics, Molecular Imaging Research Center (MIRC), Harbin Medical University, Harbin, China; ^2^Department of Nuclear Medicine, The Fourth Hospital of Harbin Medical University, Harbin, China

**Keywords:** reactive oxygen species, nanomaterials, cancer therapy, nanomedicine, nanocarriers

## Abstract

Nanotechnology advances in cancer therapy applications have led to the development of nanomaterials that generate cytotoxic reactive oxygen species (ROS) specifically in tumor cells. ROS act as a double-edged sword, as they can promote tumorigenesis and proliferation but also trigger cell death by enhancing intracellular oxidative stress. Various nanomaterials function by increasing ROS production in tumor cells and thereby disturbing their redox balance, leading to lipid peroxidation, and oxidative damage of DNA and proteins. In this review, we outline these mechanisms, summarize recent progress in ROS-based nanomaterials, including metal-based nanoparticles, organic nanomaterials, and chemotherapy drug-loaded nanoplatforms, and highlight their biomedical applications in cancer therapy as drug delivery systems (DDSs) or in combination with chemodynamic therapy (CDT), photodynamic therapy (PDT), or sonodynamic therapy (SDT). Finally, we discuss the advantages and limitations of current ROS-mediated nanomaterials used in cancer therapy and speculate on the future progress of this nanotechnology for oncological applications.

## Introduction

Cancer is the most prevalent non-communicable disease worldwide and the primary public health burden in industrialized countries (Bray et al., 2018; Siegel et al., 2019). Traditional cancer treatment strategies such as radiotherapy, chemotherapy, surgical operation, and immunotherapy present several disadvantages, including drug resistance, toxicity, high costs, and low response rates (Hainfeld et al., 2008; Hanahan and Weinberg, 2011; Kaiser, 2011; Aly, 2012; Chen et al., 2019). Recently, with the development of nanotechnology in cancer therapy, a variety of nanomaterials have been designed for malignancy treatment (Wang et al., 2008; Thakor and Gambhir, 2013). The characteristics of these nanomaterials typically depend on the distinctive features of tumors, and they target pathways underpinning the “hallmarks of cancer” (Hanahan and Weinberg, 2011). Reactive oxygen species (ROS) have been regarded as critical factors causing a range of these hallmarks (Cairns et al., 2011).

ROS are small molecules formed upon incomplete oxygen reduction, including hydrogen peroxide (H_2_O_2_), superoxide (${\text{O}}_{2}^{•-}$), singlet oxygen (^1^O_2_), and hydroxyl radical (•OH) (Trachootham et al., 2009; Gligorovski et al., 2015; Hayyan et al., 2016; Nosaka and Nosaka, 2017). Intracellular low ROS levels can regulate biological activities such as protein activation or inhibition, DNA mutagenesis, gene transcription activation, and antimicrobial activity (D'Autréaux and Toledano, 2007; Ray et al., 2012; Nathan and Cunningham-Bussel, 2013). However, high ROS levels, called oxidative stress, cause several serious diseases, such as cardiovascular diseases, cancer, neurodegenerative diseases, inflammation, and diabetes (Ide et al., 2001; Emerit et al., 2004; Valko et al., 2006; Fraisl et al., 2009; Trachootham et al., 2009).

Recent evidence shows that cancer tissues have higher ROS levels when compared to normal cells, leading to cancer proliferation and therapy resistance. Furthermore, it is well-established that ROS accumulation is linked to cellular deterioration (Gurer-Orhan et al., 2018; Cordani et al., 2020). These findings have inspired the development of methods to increase intracellular ROS concentration or to disturb cellular redox balance for cancer treatment, as tumor cells are more sensitive to excessive ROS-mediated damage (Gorrini et al., 2013; Panieri and Santoro, 2016; Zou et al., 2017).

In the past decades, nanotechnology has driven remarkable progress in medicine due to the excellent properties of nanomaterials, such as good biocompatibility, favorable pharmacological parameters, intrinsic targeting properties, and optimal physical and chemical properties (Wu and Yang, 2017; He et al., 2019; Huyan et al., 2020; Kouhpanji and Stadler, 2020). Among these novel nanoplatforms, ROS-based nanomaterials induce intracellular ROS production by targeting ROS metabolic processes, ultimately causing tumor cell death (Yang C. T. et al., 2018; Martínez-Torres et al., 2019; Li Z. et al., 2020). ROS-producing nanoparticles (NPs) can be activated through a variety of methods, including photodynamic therapy (PDT), sonodynamic therapy (SDT), chemodynamic therapy (CDT), or directly delivered chemicals that induce ROS production in cells. CDT is triggered by the Fenton reaction mainly using endogenous H_2_O_2_ and metal ions, such as iron and copper, rather than exogenously introduced energy (Lin et al., 2018; Tang et al., 2019; Wang et al., 2019; Wang W. et al., 2020). PDT can be induced by an internalized photosensitizer (PS) agent excited by light irradiation, while SDT is triggered by highly penetrating acoustic waves, which activate a class of sound-responsive sonosensitizers (Agostinis et al., 2011; Rajora et al., 2017; Li et al., 2018; Liang et al., 2020; Lin et al., 2020; Li X. et al., 2020; Lo et al., 2020). These therapeutic modalities can induce the transfer of electrons to the surrounding environment and generate ROS. Here, we review current nanomaterials used to treat cancer *via* various mechanisms and provide insights into their unique redox properties. ROS-based nanomaterials consist of metal-based NPs such as gold, iron, cerium, copper, or titanium, organic nanomaterials used as PSs or sonosensitizers, small molecules, and nanoscale drug delivery systems (DDSs) that deliver encapsulated chemical drugs.

## Metal-Based Nanoparticles for Cancer Therapy

### Gold-Based Nanomaterials

**Figure 1:**
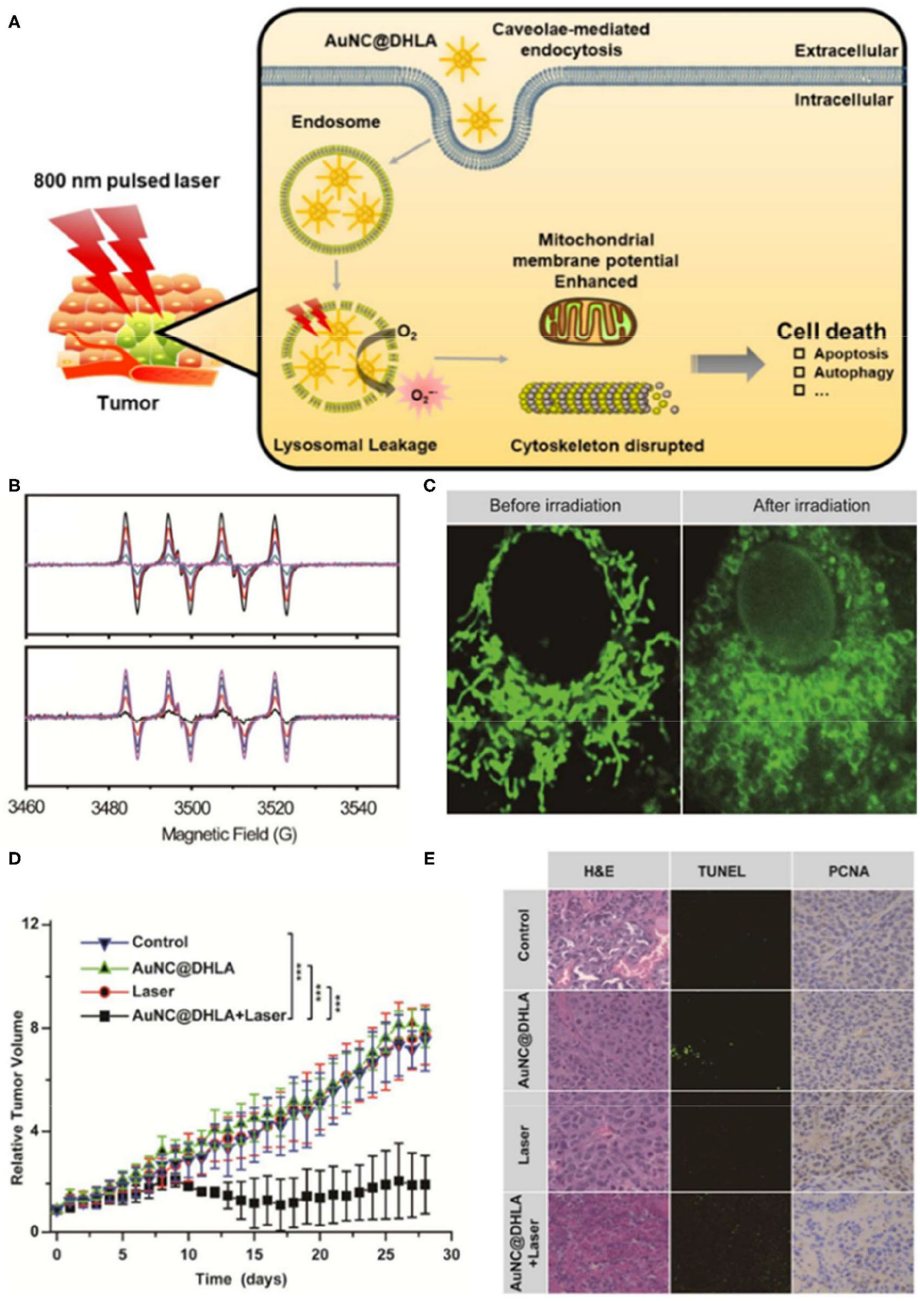
Schematic illustration of the cancer therapy mechanism **(A)** of dihydrolipoic acid-coated gold nanocluster (AuNC@DHLA) photodynamic therapy (PDT). **(B)** The Electron spin resonance (ESR) signal of ${\text{O}}_{2}^{-•}$ under 488 nm light with various power intensities and different laser irradiation durations. **(C)** Mitochondrial morphology was altered in HepG2 cells before and after irradiation. **(D)** Tumor growth curve of HepG2 tumor-bearing nude mice in different treatment groups within 28 days. **(E)** The slices of H&E staining containing liver, kidney, and spleen of tumor-bearing mice treated with phosphate-buffered saline (PBS) and AuNC@DHLA after laser irradiation treatment over a period of 28 days. Reproduced with permission from Han et al. (2020). Copyright (2019) American Chemical Society. ***p < 0.001.

Gold nanoparticles (AuNPs) are widely used in ROS-based tumor therapeutics due to their high stability, unique optical properties, and biosafety (Ashraf et al., 2016; Rogowski et al., 2016; Ding et al., 2020; Slesiona et al., 2020). AuNPs can be used as a redox catalyzer to enhance electron transfer of various electroactive biological species, thereby increasing lipid peroxidation and ROS levels, and ultimately inducing DNA double-strand breaks and cell apoptosis. Furthermore, AuNPs can also be utilized as photosensitizers for PDT cancer therapy, however, with unsatisfactory treatment outcomes. Indeed, PDT shows limited tissue penetration due to its excitation range and the intrinsic hypoxic microenvironment of solid tumors, particularly in inner regions or large tumors (Imanparast et al., 2018; Yang Y. et al., 2018; Mokoena et al., 2019). To improve the efficacy of PDT cancer therapy, Han et al. (2020) developed an intravital PDT system based on dihydrolipoic acid-coated gold nanocluster as PSs (AuNC@DHLA). AUNC@DHLA exhibited excellent two-photon optical uptake and superior photodynamic performance (Figure 1). These gold platforms generate superoxide anions through electron transfer, significantly elevating PDT efficacy in triggering cell death under hypoxia conditions (Han et al., 2020).

In addition to their superior optical characteristics, as one of high-Z elements, gold nanoclusters are promising radiosensitizers because of their ultrasmall size and robust ability to adsorb, scatter, and re-emit irradiation (Haume et al., 2016; Her et al., 2017; Laprise-Pelletier et al., 2018). To maximize the radiation dose administered to tumor cells while reducing damage to normal cells, considerable research efforts have been invested into developing effective gold nanocluster-based radiosensitizers. However, precisely correlating radiosensitizer properties with the NP inner core structure and ligands remains challenging. To overcome these long-standing challenges, AuNPs with an atomically precise structure have been designed. Jia et al. (2019) synthesized a structurally defined gold-levonorgestrel nanocluster with bright luminescence that produces ROS under X-ray irradiation, resulting in cell apoptosis. Based on excellent surface modification and water solubility by levonorgestrel for good biocompatibility, ultrasmall AuNCs that can be easily enriched in tumor areas and concentrate passing radiation, resulting in cell apoptosis. Therefore, new strategies that can design smaller radiosensitizers at the atomic level and analyze deeply the structure–activity relationship shall be explored.

### Iron-Based Nanomaterials

**Figure 2:**
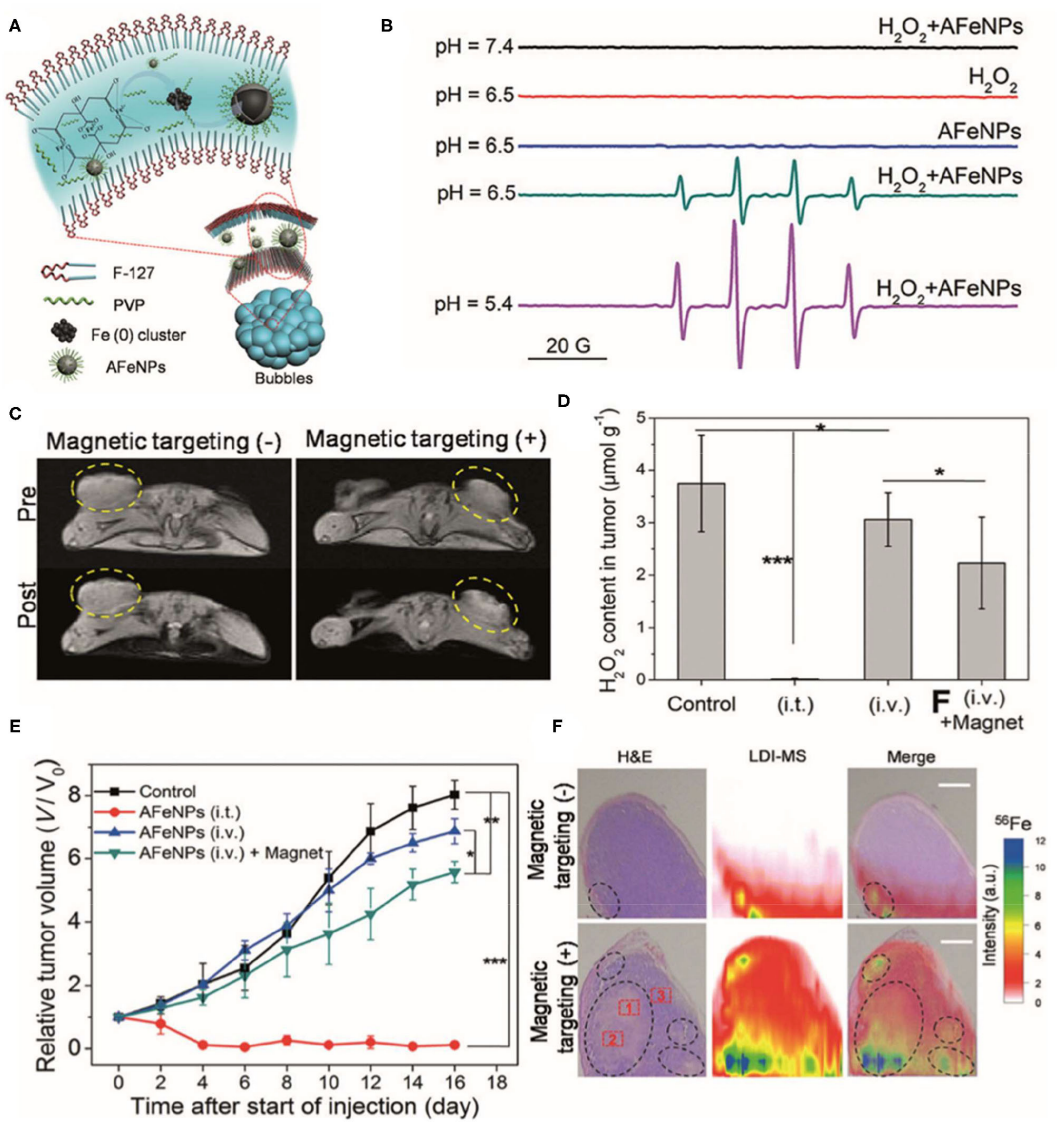
**(A)** Schematic illustration of the synthetic procedure for amorphous iron nanoparticles (AFeNPs). **(B)** ESR spectra under different conditions using 5, 5-Dimethyl-1-pyrroline N-oxide (DMPO) as the spin trap. **(C)** T_1_-weighted MRI of 4T1 tumor-bearing mice before and after intravenous administration of AFeNPs with or without the assistance of external magnetic targeting. **(D)** The hydrogen peroxide (H_2_O_2_) concentration within the tumor after either intratumoral or intravenous administration of AFeNP. **(E)** The tumor growth curve after different treatments. **(F)** H&E staining images and laser desorption/ionization mass spectroscopy (LDI-MS) ^57^Fe ^56^Fe mapping images of the tumor tissue after the intravenous administration of AFeNP with or without magnetic targeting guidance. Reproduced with permission from Zhang et al. (2016). Copyright (2016) Wiley-VCH. *p < 0.05, **p < 0.01, ***p < 0.001.

Iron oxide nanoparticles (IONPs) are the most commonly used nanomaterials in nanomedicine due to their superior biocompatibility. In addition to their superparamagnetism properties, IONPs are also thought to have excellent ROS-generating abilities based on Fenton and Haber–Weiss reactions (Winterbourn, 1995; Kehrer, 2000). Through these reactions, iron ions lead to the production of highly reactive hydroxyl or hydroperoxy radicals, thereby inducing oxidative stress and ultimately promoting DNA damage and lysosomal or mitochondria malfunction (Gaharwar et al., 2017; Sang et al., 2019; Ghosh et al., 2020; Wang X. S. et al., 2020). Studies have shown that IONPs can catalyze substrates oxidized under acidic solutions accompanied with H_2_O_2_, where IONPs show enzyme-like activity. The catalytic activity of IONPs with H_2_O_2_ generates highly toxic hydroxyl radicals (•OH), which can be used in cancer therapy. For example, Zhang et al. (2016) reported a facile synthesis of amorphous iron nanoparticles (named AFeNPs) and their superior physicochemical properties compared to their crystalline counterpart. Results showed that amorphism Fe^0^ NPs (Figure 2) can be used for cancer theranostics by inducing a Fenton reaction in the tumor and have remarkable therapeutic efficacy by acting as acidity-triggered nanocatalysts, and subsequent H_2_O_2_ disproportionation leads to efficient •OH generation to induce tumor CDT (Zhang et al., 2016).

Inspired by these observations, Yu et al. (2019) investigated the interaction of IONPs with H_2_O_2_ *in vitro* and *in vivo*, aiming to improve ROS therapeutic efficacy. Core-shell-structured iron carbide (Fe_5_C_2_@Fe_3_O_4_) NPs were synthesized, with the nanostructures occupying the interstitial spaces between the iron lattice. This structure provides higher stability to the NPs in oxygenated/hydrous-containing atmospheres, thereby improving biocompatibility and life span. Notably, these core-shell NPs are more sensitive to acidity and can discharge ferrous ions more effectively in low-pH environments with a resulting overproduction of H_2_O_2_ in tumor regions, which in turn induces ROS production. Thus, Fe_5_C_2_@Fe_3_O_4_ can be used for effective tumor-targeted therapy with higher safety (Yu et al., 2019). Interestingly, in addition to its CDT effects, recent studies on IONPs have also revealed the sensitizing effect of radiotherapy. While the exact mechanisms remain unclear, several reports have shown that radiation can enhance mitochondrial respiration and, combined with IONPs, generate remarkably high levels of ROS in cancer cells (Hauser et al., 2016; Klein et al., 2018).

Expect of the applications in the field of CDT, IONPs-based ferroptosis and immunotherapy had been reported recently; those were based on ROS as well (Dixon et al., 2012; Zanganeh et al., 2016; Yu et al., 2020). A new cancer immunotherapy that activates macrophages by IONPs has been reported. Zanganeh et al. (2016) made a breakthrough in the study of ferumoxytol [approved by the Food and Drug Administration (FDA) for clinical use]. Studies have shown that injection of ferumoxytol in mouse can inhibit the growth of early breast cancer and prevent metastasis of lung cancer. In addition, ferumoxytol can stimulate tumor-related macrophages to differentiate into M1-activated type and secrete pro-inflammatory factors and induce tumor cell apoptosis by activating the caspase-3 pathway (Zanganeh et al., 2016).

### Cerium-Based Nanomaterials

Cerium oxide NPs are commonly used rare-earth nanomaterials with high chemical reactivity due to the catalytic activity of Ce^3+^ and Ce^4+^ on the surface of the particles. Cerium-based NPs have been successfully used in the treatment of various cancers *in vivo* and *in vitro* (Pešić et al., 2015; Baskar et al., 2018; Adebayo et al., 2020). Studies have shown that cerium oxide NPs can improve wound healing, reduce nerve cell death, and inhibit the growth of tumor cells by increasing ROS levels (Hijaz et al., 2016; Xiao et al., 2016; Naz et al., 2017). The unique redox surface chemical properties of cerium oxide NPs give them the ability to promote both antioxidation and oxidation, which, combined with low toxicity in wild-type cells, opens the possibility for a wide range of clinical applications (Wason et al., 2013; Rzigalinski et al., 2017).

Cerium oxide NPs exhibit antitumor activity based on oxidative stress and apoptotic effects without damaging normal cells. For example, Nourmohammadi et al. (2019) showed that nanoceria promote ROS production and apoptosis in a dose-dependent manner in fibrosarcoma tumor cells *in vitro* but have no impact on normal cells even at extremely high concentrations. In a subsequent study, the authors demonstrated by real-time PCR and Western blot that nanoceria significantly improved Bax expression in fibrosarcoma tumor cells, thus confirming their antitumor efficacy (Nourmohammadi et al., 2020). These findings suggest that cerium oxide NPs are a promising treatment strategy for fibrosarcoma.

**Figure 3:**
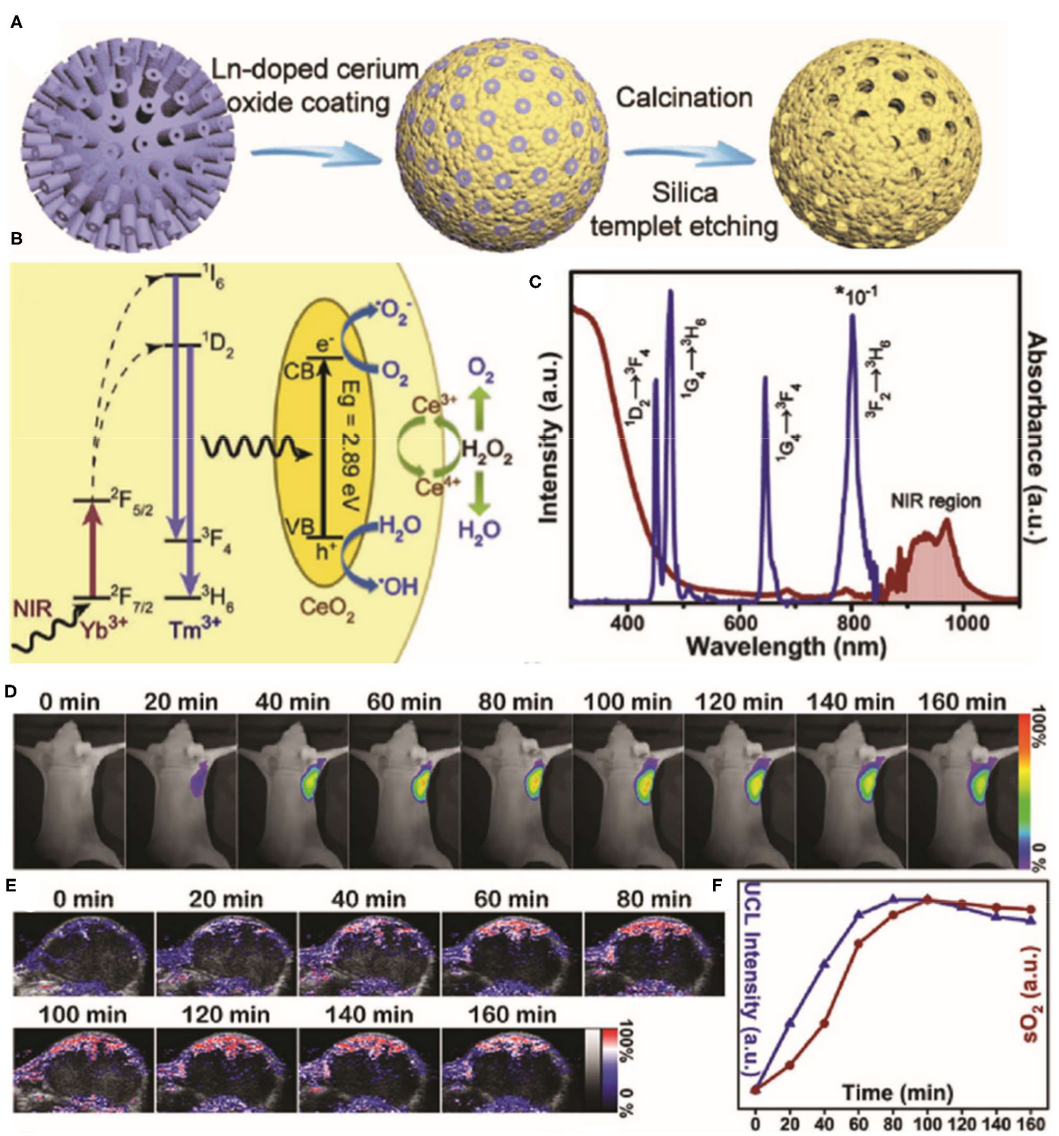
Schematic illustration of the synthesis process **(A)** for the Yb^3+^/Tm^3+^ codoped mesoporous cerium oxide hollow nanoparticles [cerium oxide upconversion nanoparticles (Ce-UCNPs)]. **(B)** Schematic illumination of the upconversion, photocatalysis, and enzyme-like catalysis mechanism of the Ce-UCNP (from left to right). **(C)** The UV–vis–IR Diffuse reflectance spectra (DRS) and Upconversion luminescence (UCL) of the Ce-UCNPs. **(D)** Upconversion bioimaging signals of Ce-UCNPs in the U87MG tumor regions. **(E)** Representative photoacoustic (PA) images of solid tumors by measuring oxygenated hemoglobin after injection of Ce-UCNPs at various time points. **(F)** Quantification of the upconversion bioimaging and the blood oxygen saturation signals in **(D,E)**. Reproduced with permission from Yao et al. (2018). Copyright (2018) Wiley-VCH. *p < 0.05.

The therapeutic efficacy of oxygen-dependent PDT and drug-based chemotherapy is limited by the hypoxic conditions in tumors. Given their antioxidation properties, cerium oxide NPs may be used to deliver oxygen to tumor regions. Indeed, it was shown that cerium oxide NPs generate O_2_ inside tumors by consuming endogenous H_2_O_2_ (Yao et al., 2018; Liu et al., 2020; Zeng et al., 2020). For example, Yao et al. (2018) reported that a synergetic mesoporous cerium oxide upconversion nanoparticle (Ce-UCNP; Figure 3) achieves endogenous H_2_O_2_-responsive O_2_ self-sufficiency and near-infrared (NIR) light-controlled PDT simultaneously. Firstly, Ce-UCNPs supplied sufficient O_2_ by decomposing endogenous H_2_O_2_ in tumor through enzyme-like catalysis, which enhanced PDT in neutral and acidic microenvironments. While under NIR laser irradiation, UCNPs can convert NIR to UV source and trigger the photocatalysis reaction catalyzed by cerium oxide, produce ${\text{O}}_{2}^{•-}$ and •OH to trigger cellular apoptosis. In addition, the Ce-UCNPs with specific mesoporous hollow nanostructure and Arg-Gly-Asp (RGD)-peptide surface modification can also act as a delivery cargo to realize pH-responsive doxorubicin (DOX) release in the acid tumor site and organelles of α_v_β_3_ integrin-rich cancer cells for chemotherapy.

### Copper-Based Nanomaterials

Catalytical medicine is a unique curative strategy with accurate disease specificity and low systemic side effects, whereby nanocatalysts mediate specific chemical reactions in the body. Cu-based Fenton reactions convert H_2_O_2_ into toxic hydroxyl radicals across a wide pH range more rapidly and effectively than Fe-based Fenton reactions, making Cu-based nanomaterials more versatile and affordable. Specifically, the reduction rate of Cu^2+^ ion by H_2_O_2_ was calculated to be 4.6 × 10^2^ M^−1^s^−1^, which was much higher than that of Fe^3+^ ion (0.001–0.02 M^−1^s^−1^). Moreover, Cu^+^ ion could catalyze H_2_O_2_ efficiently to generate •OH radicals with a higher reaction rate (1 × 10^4^ M^−1^s^−1^) than that of Fe^2+^ ion (76 M^−1^s^−1^) (Perez-Benito, 2004; Hu et al., 2019). Thus, different Cu-based Fenton nanotherapeutics have been explored for oxidative cancer treatment. Hu et al. (2019) reported that Cu-based nanocatalysts significantly outperform most of the investigated Fe-based nanocatalysts. In particular, the novel ultrasmall (≤5 nm) PEGylated Cu_2−x_S nanodots leaded that photoacoustic (PA) imaging and photothermal therapy in a light-activated photonic theranostic modality of NIR-II biowindow (1,000–1,350 nm) could accurately measure tumor area and increase Fenton-induced treatment by photonic hyperthermia. These results demonstrated the curative effect of Cu_2−x_S-enabled interactive photothermal hyperthermia-enhanced nanocatalytic therapy. This research not only puts forward a new combined NP/photothermal therapy synergistic cancer treatment model but also expands the nanocatalyst repertoire for Fenton reaction-based cancer therapy.

### Titanium-Based Nanomaterials

Titanium-based nanoparticles (TiNPs) have recently raised interest in a large number of biomedical applications. TiNPs are excellent photosensitizers for PDT cancer treatment due to their outstanding ROS-generating ability when exposed to light sources (Rehman et al., 2016; Lan et al., 2019; Ziental et al., 2020). Recently, studies showed that TiNPs can enhance oxidative stress and DNA damage in tumor cells as a result of increased catalytic activity, particularly in the range of 1–100-nm diameter (Li et al., 2020).

**Figure 4:**
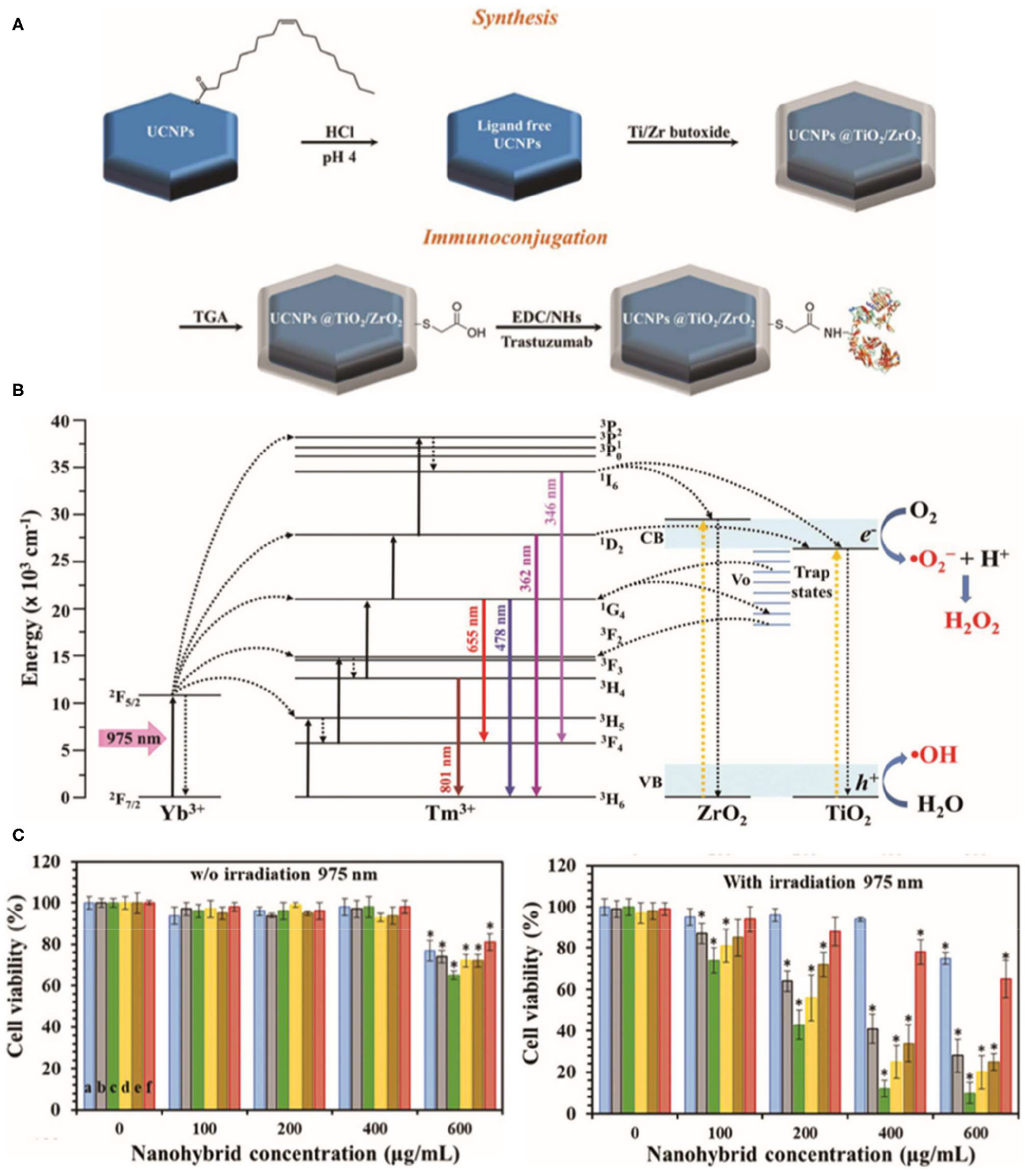
Schematic illustration of the synthesis of the NaYF_4_:Yb,Tm@TiO_2_/ZrO_2_ core@shell nanocomplex **(A)** and bioconjugation with trastuzumab. **(B)** Energy-level diagram of the upconversion, energy transfer, and reactive oxygen species (ROS) production processes in the NaYF_4_:Yb,Tm@TiO_2_/ZrO_2_ nanocomplex under 975 nm excitation. **(C)** alamarBlue assay to assess the cell viability of SKBR3 cells incubated with various nanocomplex concentrations without light exposure and upon 975 nm continuous laser irradiation (0.71 W cm^−2^) during 5 min. Reproduced with permission from Ramírez-García et al. (2019). Copyright (2019) Royal Society of Chemistry. *p < 0.05.

The ROS-based cytotoxic antitumor effects of TiNPs have been demonstrated in various tumor models (Cheng et al., 2018; Yang et al., 2020; Zheng et al., 2020), such as breast cancer, non-small-cell lung cancer, cervical cancer, and colon cancer. Recently, Ramírez-García et al. (2019) synthesized a photocatalytic TiO_2_/ZrO_2_ shelled UCNP that increased oxygen defects in human epidermal growth factor receptor 2 (HER2)-positive breast cancer cells. The nanoplatform enhances charge separation and reduces the rate of electron–hole pairs recombination, which is produced by resonant energy transfer from donor to acceptor, ultimately leading to increased ROS production. The hybrid photosensitizer is excited through non-radiative energy transmission (975 nm irradiation, used as the UV upconversion emission band), while NIR emission (801 nm irradiation) can be used to track cells (Figure 4). Notably, the TiNPs reduced cell viability to 12% after 5 min of continuous exposure to 975 nm light at 0.71 W cm^−2^. In addition, an added antibody provided specificity to the cancer cells. Thus, this powerful TiNP-based PDT tool can significantly reduce the survival rate of breast cancer cells with high specificity under low-energy irradiation.

## Organic Nanoparticles for Cancer Therapy

### Organic Photosensitizers and Sonosensitizer-Based Nanomaterials

Organic photosensitizers and sonosensitizers are also widely used in ROS-based nanomaterials for tumor PDT and SDT therapy, respectively. Photosensitizers are excited when exposed to light with an appropriate wavelength. Before returning to the basic state, the activated photosensitizer sends energy directly to its surroundings, which induces production of ROS (Ozog et al., 2016; Prazmo et al., 2016; Kwiatkowski et al., 2018). However, as this process requires O_2_, the efficacy of PDT is limited in the tumor hypoxic microenvironment. SDT is a powerful new treatment strategy that overcomes this problem while providing non-invasive deep tissue penetration with negligible side effects (McHale et al., 2016; Geng et al., 2018; Bilmin et al., 2019). Similar to photosensitizers in PDT, SDT sonosensitizers are activated by sonoluminescence and produce ROS.

Despite the favorable therapeutic outcomes of ROS-based therapeutics, cell autophagy may be activated by ROS, which blocks apoptosis of tumor cells. To address this, Deng et al. (2020) designed an innovative supramolecular nanoplatform based on photosensitizer Chlorin e6 (Ce6)-encapsulated NPs containing respiration inhibitor 3-bromopyruvate (3BP), which can act as an autophagy promoter and hypoxia ameliorator, reducing intracellular oxygen consumption rate. Results showed that the Ce6-3BP nanoplatform overcomes the solubility defect of Ce6 and improves pharmacokinetics and accumulation of NPs in tumor sites. Excessive autophagy triggered by the combination of ROS and starvation converted the role of autophagy from pro-survival to pro-death, further promoting cell apoptosis. Therefore, enhanced PDT could be achieved by 3BP-induced autophagy and hypoxia relief, resulting in sufficient PDT effect. In another report, Wang et al. (2011) showed that a UCNP-Ce6 supramolecular complex with Ce6 loaded onto the entangled skeleton of the hydrophobic alkyl chains of amphiphilic C_18_ MPH–PEG molecules provides good PDT and antitumor growth effects.

Multifunctional NPs that integrate diagnosis and treatment are highly desirable in precision medicine. PA imaging-guided sonosensitizers can be used to precisely kill tumor cells and tissue. Huang et al. (2018) developed distinctive core/shell-structured theranostic organic hematoporphyrin-based nanoparticles (FHMP NPs) with broad optical absorption. These NPs showed excellent PA imaging contrast enhancement properties and significantly improved SDT efficacy. Notably, this Poly(lactic-co-glycolic acid) (PLGA)-based nanoplatform increased light-source constancy of Hematoporphyrinmonomethyl Ether (HMME), increased sonodynamic capacity, and enhanced the delivery of NPs to the tumor site. Furthermore, a synergistic action between HMME and melanin NP was found and validated. Finally, the ultrasound-triggered sonosensitizer accelerated ROS-induced cytotoxicity of tumor cells. Thus, FHMP NPs are a promising ROS-based SDT nanoplatform for inhibition of tumor proliferation with excellent biosafety (Huang et al., 2018).

## Nanomaterials as Drug Delivery System for Cancer Therapy

Over the past decades, it has been demonstrated that targeted drug delivery combined with intracellular ROS production improves the efficacy of chemotherapeutic agents, such as paclitaxel, cinnamaldehyde, camptothecin, cisplatin, β-Lapachone, docetaxel, and so on. These anticancer drugs are often used as amplification agents of oxidative stress to produce ROS, leading to preferential killing of cancer cells *in vitro* and *in vivo* (Noh et al., 2015; Feng et al., 2020). The potential reasons were that chemotherapeutic drugs can generate ROS through catalyzing intracellular enzymes like NAD(P)H:quinone oxidoreductase-1 (NQO1) and nicotinamide adenine dinucleotide phosphate (NADPH) oxidase (Ye et al., 2017; Dai et al., 2018). A vast array of DDSs had been designed by changing drug pharmacokinetics and biodistribution to improve therapeutic efficacy (Ashfaq et al., 2017; Unsoy and Gunduz, 2018). However, extensive administration of DDSs can cause toxicity due to their poor metabolism and elimination. With the development of nanomedicine, drug-loaded nanocarriers have been proven suitable for single- and multi-drug delivery treatments (Liang and Liu, 2016; Yang et al., 2016; Feng et al., 2019; Handali et al., 2019).

Yoo et al. (2018) developed a polysaccharide-based DDS with powerful treatment efficacy and high drug loading, potentially allowing for the reduction of drug dosage. The authors used maltodextrin combined with cinnamaldehyde, the primary constituent of cinnamon, to increase intratumoral ROS production. Cinnamaldehyde is easy to synthesize and encapsulate by linking to the hydroxyl groups of maltodextrin *via* acid-cuttable acetal connections. These cinnamaldehyde-combined maltodextrin (CMD) NPs mediated acid-induced ROS production, resulting in apoptotic cell death. The anticancer drug camptothecin (CPT) was selected to assess the synergistic antitumor efficacy of CMD NPs. CPT-loaded CMD NPs showed stronger antitumor effects than empty CMD NPs or CPT alone in mouse xenograft tumor models, demonstrating the synergistic effect of CMD with CPT in cancer therapy. Thus, CMD NPs are polymeric prodrugs of cinnamaldehyde and drug delivery carriers with great potential against malignancy (Yoo et al., 2018).

Cancer still ranks first in diagnosis and mortality in the world, despite substantial progress in oncology, biomedicine, and drug research and development. Although anticancer drugs used in traditional chemotherapy are still widely used to reduce tumor burden in patients, they can present multiple disadvantages depending on their chemical structure and on their destination after intravenous delivery. Noh et al. (2019) developed a promising oxidation-based strategy to address these challenges by combining cinnamaldehyde (a ROS-generating compound) with zinc protoporphyrin (an antioxidant inhibiting agent) to proton-responsive polymers. On the one hand, concurrent delivery of both therapeutic agents and high drug loading capacity enhanced bioavailability, and on the other, disassembly of the micelles and cargo release specifically in the tumor microenvironment low pH conditions increased tumor cell oxidative stress, ultimately resulting in high levels of apoptosis (Noh et al., 2019).

## Summary and Outlook

In this review, we have succinctly described various types of nanomaterials based on ROS applied in cancer therapy. The application of ROS-based NPs in nanomedicine and preclinical research has made great advances and currently provides a solid foundation for further clinical translation. ROS-based nanomaterials increase intracellular oxidative stress in combination with treatment methods such as PDT, SDT, CDT, or loaded redox drugs. However, although nanotechnology strategies in cancer therapy have shown encouraging results in preclinical research and clinical trials, several important challenges need to be addressed to successfully translate ROS-based nanomaterials to the clinic:

(1) The importance of pharmacokinetics after NP injection has become evident with the development of nanotechnology in cancer treatment. To improve therapeutic efficacy, researchers have mostly focused on NP accumulation in the tumor site and lost sight of the potential side effects to healthy tissues due to long-term retention. On the other hand, although rapid clearance from the body lessens cytotoxicity, it inevitably leads to low concentration and accumulation of the therapeutic NPs in the target location. This dilemma remains a challenge in nanotechnology and should be addressed in future research.
(2) Although numerous studies have demonstrated the antitumor therapeutic efficacy of ROS-based nanomaterials in *in vitro* and *in vivo* models, mechanistic insights are still lacking. To date, the majority of ROS-based treatment strategies have only focused on therapeutic outcomes and failed to explain the underlying molecular mechanisms. Such knowledge would help design ROS-based NPs harmless to normal environments and thus minimize adverse clinical secondary effects.
(3) Multiple nanodelivery systems have taken advantage of the enhanced permeability and retention (EPR) effect with undisputable success in small-animal subcutaneous xenograft tumors. More recent research, however, has revealed that the EPR effect is responsible for < 1% delivery efficiency, and in orthotopic transplants and larger species, this efficiency has been shown to be even lower. Furthermore, intravenous NP delivery may be ineffective due to untargeted uptakes and elimination from the body before effective accumulation in target sites. Thus, alternative delivery systems, such as oral administration, subcutaneous injection, intratracheal delivery, and potential novel strategies may open new avenues for NP applications and therapeutic strategies in living organisms.

## Author Contributions

YL and JY wrote the manuscript. XS revised the manuscript. All authors contributed to the article and approved the submitted version.

## Conflict of Interest

The authors declare that the research was conducted in the absence of any commercial or financial relationships that could be construed as a potential conflict of interest.
